# Bacteriophage ΦSA012 Has a Broad Host Range against *Staphylococcus aureus* and Effective Lytic Capacity in a Mouse Mastitis Model

**DOI:** 10.3390/biology7010008

**Published:** 2018-01-09

**Authors:** Hidetomo Iwano, Yusuke Inoue, Takuji Takasago, Hironori Kobayashi, Takaaki Furusawa, Kotomi Taniguchi, Jumpei Fujiki, Hiroshi Yokota, Masaru Usui, Yasunori Tanji, Katsuro Hagiwara, Hidetoshi Higuchi, Yutaka Tamura

**Affiliations:** 1 Laboratory of Veterinary Biochemistry, School of Veterinary Medicine, Rakuno Gakuen University, Ebetsu, Hokkaido 069-8501, Japan; y.inoue.85@city.nagoya.lg.jp (Y.I.); takasago.wa8@pref.kanagawa.jp (T.T.); hiro4kjp@yahoo.co.jp(H.K.); s21441012@stu.rakuno.ac.jp (T.F.); kotomi.taniguchi@elsakreis.jp (K.T.); j-fujiki@rakuno.ac.jp (J.F.); h-yokota@rakuno.ac.jp (H.Y.); 2 Laboratory of Food Microbiology and Food Safety, School of Veterinary Medicine, Rakuno Gakuen University, Ebetsu, Hokkaido 069-8501, Japan; usuima@rakuno.ac.jp (M.U.); tamuray@rakuno.ac.jp (Y.T.); 3 Department of Bioengineering, Tokyo Institute of Technology, Yokohama 226-8502, Japan; ytanji@bio.titech.ac.jp; 4 Laboratory of Veterinary Virology, School of Veterinary Medicine, Rakuno Gakuen University, Ebetsu, Hokkaido 069-8501, Japan; k-hagi@rakuno.ac.jp; 5 Laboratory of Veterinary Hygiene, School of Veterinary Medicine, Rakuno Gakuen University, Ebetsu, Hokkaido 069-8501, Japan; higuchi@rakuno.ac.jp; 6 Center for Veterinary Drug Vevelopment, Rakuno Gakuen University, Rakuno Gakuen University, Ebetsu, Hokkaido 069-8501, Japan

**Keywords:** bacteriophage, mastitis, *Staphylococcus aureus*

## Abstract

Bovine mastitis is an inflammation of the mammary gland caused by bacterial infection in dairy cattle. It is the most costly disease in the dairy industry because of the high use of antibiotics. *Staphylococcus aureus* is one of the major causative agents of bovine mastitis and antimicrobial resistance. Therefore, new strategies to control bacterial infection are required in the dairy industry. One potential strategy is bacteriophage (phage) therapy. In the present study, we examined the host range of previously isolated *S. aureus* phages ΦSA012 and ΦSA039 against *S. aureus* strains isolated from mastitic cows. These phages could kill all *S. aureus* (93 strains from 40 genotypes) and methicillin-resistant *S. aureus* (six strains from six genotypes) strains tested. Using a mouse mastitis model, we demonstrated that ΦSA012 reduced proliferation of *S. aureus* and inflammation in the mammary gland. Furthermore, intravenous or intraperitoneal phage administration reduced proliferation of *S. aureus* in the mammary glands. These results suggest that broad host range phages ΦSA012 is potential antibacterial agents for dairy production medicine.

## 1. Introduction

Bovine mastitis is the most prevalent disease, which is defined as inflammation of the udder, commonly caused by bacterial infection in dairy cattle [1]. It is the most costly disease in the dairy industry because of the high use of antibiotics [2]. *Staphylococcus aureus* is a Gram-positive pathogen that is involved in a variety of infectious diseases in human and animals [3]. *S. aureus* is also one of the most frequent causative agents of subclinical and clinical bovine mastitis with prevalence rates as high as 50% in some countries [4], resulting in the high use of antibiotics. Antimicrobial resistance in bacteria is a concern not only in veterinary medicine, but also in general worldwide [5,6]. In 1928, penicillin was developed and subsequently used globally. However, methicillin-resistant *S. aureus* (MRSA), vancomycin-resistant enterococci, multidrug-resistant *Pseudomonas aeruginosa*, and other drug-resistant bacteria have recently emerged [7]. These bacteria can put the lives of humans and animals at great risk; for instance, MRSA is especially widespread in human hospitals. Furthermore, MRSA carriage is potentially an occupational risk for veterinary personnel [8]. When antibiotic resistance first emerged, novel antibiotics to treat these bacteria were developed. However, it is difficult to develop effective new antibiotics every time that a new resistance mechanism emerges. Moreover, many pharmaceutical companies are now withdrawing from the antimicrobial development business. Therefore, proper use of currently available antibiotics is necessary to prevent selecting for multidrug-resistant bacteria. Furthermore, there is a demand for new strategies to treat bacterial infections, such as antagonistic bacteria, antimicrobial peptides, phage therapy, phage endolysins, gene-editing enzymes, and metals [9,10].

Phage therapy is particularly promising as it involves a bacteriophage (phage) that specifically infects and kills bacteria. Phages were co-discovered by Felix d’Herelle and Frederick Twort [11], and phage therapy was proposed by d’Herelle in 1931 [12]. Many phages that were isolated previously are classified into the order *Caudovirales* [13]. After their discovery, the use of phages for therapy had been studied in European countries, but waned because of the success of penicillin. Nevertheless, phages have been used in the former Soviet Union and Eastern Europe to the present day [11,14]. Indeed, researchers have reported the efficacy of phage therapy for experimental bacterial disease [7,15,16,17,18]. Examples include applications for the use of the phages ListShield (Intralytix, Inc., Baltimore, MD, USA) for *Listeria monocytogenes* and EcoShield (Intralytix, Inc.) for *Escherichia coli* O157:H7 to protect against foodborne disease [19,20]. In addition, not only phage, but also phage endolysin, which is used at the end of the phage lytic cycle, is being assessed for therapeutic use [17,21]. The quick approval of more applications of phage therapy for bacterial infections that are hard to treat is expected. Investigations of phage therapy for bovine mastitis have been reported [18,22,23]; however, further study is necessary for it to be established as an effective therapeutic antibacterial agent. Recently, we reported new *S. aureus* phages isolated from sewage samples with broad lytic activity against *S. aureus* strains [24]. We consider that these phages may be used for phage therapy against various human and livestock diseases.

In the present study, we demonstrated that *S. aureus* phage ΦSA012 has a broad host range against *S. aureus* strains, causing bovine mastitis. Furthermore, using a mouse model of bacterial mastitis, we showed the effectiveness of different administration routes for effective phage therapy against mastitis.

## 2. Materials and Methods

### 2.1. Ethical Treatment of Animals

This study was carried out in strict accordance with the recommendations in the Guidelines for Proper Conduct of Animal Experiments, Science Council of Japan [25]. The protocol was approved by the Committee on the Ethics of Animal Experiments of Rakuno Gakuen University (approval numbers VH21A20, approved 23 April 2009, and VH22A8, approved 16 June 2010. The experiments using animals are finished until March 2011). Welfare-related assessments and interventions were carried out prior to, during, and after the experiment.

### 2.2. Animals

Healthy specific-pathogen-free 8-week-old ddY lactation mice, which had the same weight, were purchased from Sankyo Labo Service Corporation, Inc. (Tokyo, Japan) and housed under pathogen-free conditions with pups at the animal facility of Rakuno Gakuen University (Hokkaido, Japan). All of the animals were housed in a temperature-controlled room under a 12 h/12 h light-dark cycle for at least one week to acclimate to the surroundings and with free access to food and water.

### 2.3. Bacterial Strains and Culture Media

Bacterial strains were isolated from mastitic cows on dairy farms in Hokkaido, Japan. Staphylococcus was identified as the main causative agent by standard culture-based and biochemical procedures on site. All of the samples were collected in sterile tubes, kept on ice, and then stored at −20 °C during transport to the laboratory where an analysis was performed. The milk was diluted when necessary, and 100 µL was plated on sheep blood agar plates. Colonies were assayed for coagulase activity. All isolated *S. aureus* with a double-positive phenotype were purified DNA and used to determine genotype by a multiplex PCR kit (CicaGeneus Staph POT KIT) [26,27]. The POT type was calculated according to the kit manual. Luria-Bertani (LB) medium was used for bacterial or phage culture and for counting colony-forming units (CFUs) of *S. aureus* strain SA003 previously harvested from bovine milk [24]. SM buffer (10 mM MgSO_4_, 100 mM NaCl, 0.01% gelatin, and 50 mM Tris-HCl [pH 7.5]) was used for phage dilution. To assess phage plaque formation, LB medium containing 1.5% agar or 0.5% agarose ME (Iwai Chemicals Company, Tokyo, Japan) was used to form the lower and upper layers, respectively.

### 2.4. Host Specificity of Phages (Spot Test)

The procedure was carried out, according to a previously reported method [28,29]. A 5-µL aliquot of phage suspension (10^10^ plaque-forming units (PFU)/mL) was dropped onto a double-layer agar plate containing an *S. aureus* strain. The plate was then incubated at 37 °C overnight. After incubation, the infected area was characterized as one of four categories: clear plaques, turbid plaques, faint plaques, or no plaques.

### 2.5. Mouse Model of S. aureus Mastitis

*S. aureus* strain SA003, which has been reported to be susceptible to ΦSA012 [24], was grown in 6 mL of LB medium at 37 °C, and the cell number was determined by measuring the optical density at 600 nm (OD_600_) of the medium. When the density was about 2 × 10^8^ CFU/mL (around 0.5 OD_600_), the culture was centrifuged at 10,000× *g* for 5 min. The cell pellet was washed with 1 mL of phosphate-buffered saline (PBS), and then recentrifuged under the same conditions. The pellet was resuspended and diluted in PBS to a density of 2 × 10^6^ CFU/mL. Phage was isolated by large-scale culture using soft agar plate, and the phage suspension was purified by density gradient ultracentrifugation using CsCl, according to a previously reported method [29]. Mice were anesthetized 30 min before infection with a mixture of three types of anesthetic agents: 6 mg/kg midazolam (Dormicum; Astellas, Tokyo, Japan), 0.45 mg/kg medetomidine (Dorbene; Kyoritsu Seiyaku Corp., Tokyo, Japan), and 7.5 mg/kg butorphanol (Vetorpale; Meiji Seika Pharma Co., Ltd., Tokyo, Japan). Chronic experimental mastitis mice were infected using a protocol modified from that of Tuchscherr et al. [30]. Briefly, 7–10 days after parturition, lactating mothers were separated from the pups and anesthetized 4 h before infection. The left and right fourth mammary glands (L4 and R4, respectively) were injected with 25 µL of *S. aureus* suspension (10^3^ and 10^5^ CFUs) delivered through a 34-gauge needle and syringe, and subsequently injected in a similar manner with 25 µL of ΦSA012 suspension (10^5^ and 10^7^ PFUs) into the mammary gland or 100 µL of ΦSA012 suspension (4 × 10^7^ PFUs) intraperitoneally or intravenously. Two hours after challenge, the mothers were returned to the pups and remained with them for the remainder of the experiment. Groups of lactating mice were euthanized on days 2 and 4, and the L4 and R4 glands were aseptically removed. Each gland was homogenized in 2 mL of PBS, and dilutions of the homogenates were plated quantitatively to determine the number of CFUs per gland. In separate experiments, L4 and R4 mammary glands were excised for histological examination. Whole glands were fixed in 4% paraformaldehyde, dehydrated with 70–100% ethanol, cleared with xylene, and embedded in paraffin. Tissue sections were stained with hematoxylin-eosinand and observed using microscope with camera system (CKX41 and DP70 Olympus, Tokyo Japan). For experiments in which phage was administered intraperitoneally or intravenously, total DNA was purified from blood and mammary glands, and then phage genomic DNA (phiSA012; NC_023573.1) was enumerated by real-time PCR with phage-specific primers (endolysin; YP_009006743.1 F: 5′-ATGACGCTCAATCAGCTCCG-3′, R: 5′-CCGTCTTCTTGTGCATCTTTAACA-3′) compared with dilution standard of phage genomic DNA. We confirmed no amplification form a mouse tissue without phage administration.

### 2.6. Analysis of Data

All of the statistical analysis was performed using Excel statistics version 2010, for Windows. Data were analyzed using analysis variance with the Tukey multiple-comparison test. 

## 3. Results

### 3.1. Host Ranges of S. aureus Phages ΦSA012 and ΦSA039

ΦSA012 and ΦSA039 have been reported as broad-range lytic *S. aureus* phages [24]. To confirm this activity, we examined the host range of *S. aureus* phages ΦSA012 and ΦSA039 against 93 *S. aureus* strains isolated from milk of cows with *S. aureus* bovine mastitis in Kushiro (Table 1; *n* = 57) and Ishikari (Table 2; *n* = 36) in Hokkaido, Japan. To investigate clonality of *S. aureus*, multilocus sequence typing (MLST) is generally performed. However, MLST analysis can be cost prohibitive. Therefore, in this study, *S. aureus* genotypes were determined using a multiplex PCR kit (CicaGeneus Staph POT KIT). The kit was developed as a multiple sample inspection method by determining the conservation pattern of “small genomic islets”, which are nonconserved regions between strains [26,27]. The genotypes of strains analyzed in the present study were determined by the multiplex PCR kit, which detected 40 genotypes. Both ΦSA012 and ΦSA039 produced clear plaques with many *S. aureus* strains, especially indicating that ΦSA012 could kill *S. aureus* completely (Table 1 and Table 2). In addition, we showed ΦSA012 is also effective against MRSA (Table 3 and Supplemental Table S1).

### 3.2. Effect of Phage ΦSA012 in a Mouse Model of S. aureus Mastitis

**Figure 1:**
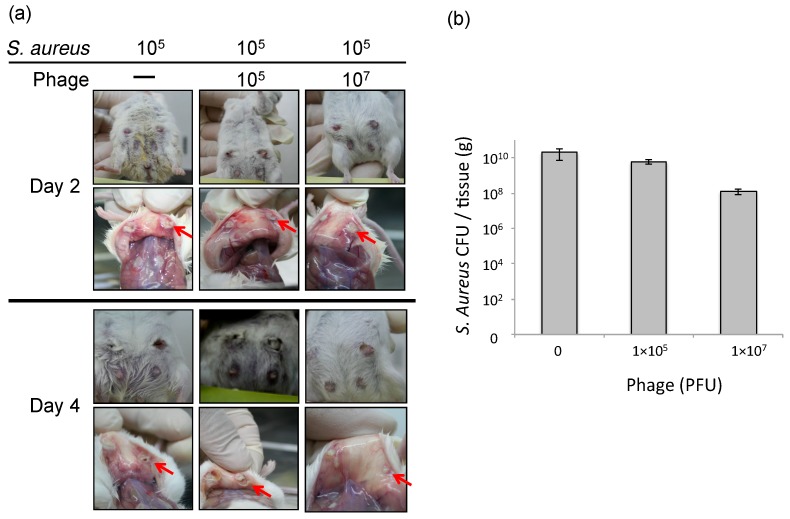
Therapeutic efficacy of ΦSA012 in a mouse model of *S. aureus* mastitis. (**a**) Photographs of the mouse mastitis model after administration of SA003 (10^5^ CFU, *n* = 5) and phage treatment (10^5^ PFU (MOI = 1, *n* = 7) and 10^7^ PFU (MOI = 100, *n* = 6)). Arrows indicates abscesses. (**b**) *S. aureus* CFUs in the mammary glands on day 2 after challenge. Bars indicate standard errors of the means. There was no statistically significant difference.

**Figure 2:**
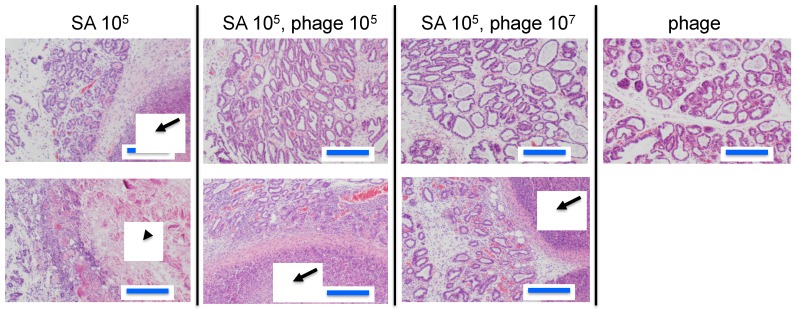
Pathological images of mammary glands in the mouse model of *S. aureus* mastitis. Arrows indicate abscesses and the arrowhead indicates an area of broken tissue. Blue bars indicate 200 µm long.

To estimate the efficacy of phage therapy against bovine mastitis, we carried out an in vivo experiment using an established mouse model of *S. aureus* mastitis using phage ΦSA012 administration through directry injection into the mammary glands [30]. ΦSA012 is more effective against broad *S. aureus* strain than ΦSA039 in spot tests. Gross examination of mammary glands showed that phage administration moderately reduced inflammation (Figure 1a). The bacterial count decreased by phage administration at a multiplicity of infection (MOI) of 100 (Figure 1b). In the pathological analysis, *S. aureus* caused strong inflammation and destroyed mammary gland structures (Figure 2). However, phage administration reduced the destruction of mammary gland structures. Administration of phage only did not result in any change in the mammary glands. These results suggest that phage administration at an MOI of 100 inhibit *S. aureus* proliferation and infection. 

### 3.3. Transport of Phage to Mammary Glands

**Figure 3:**
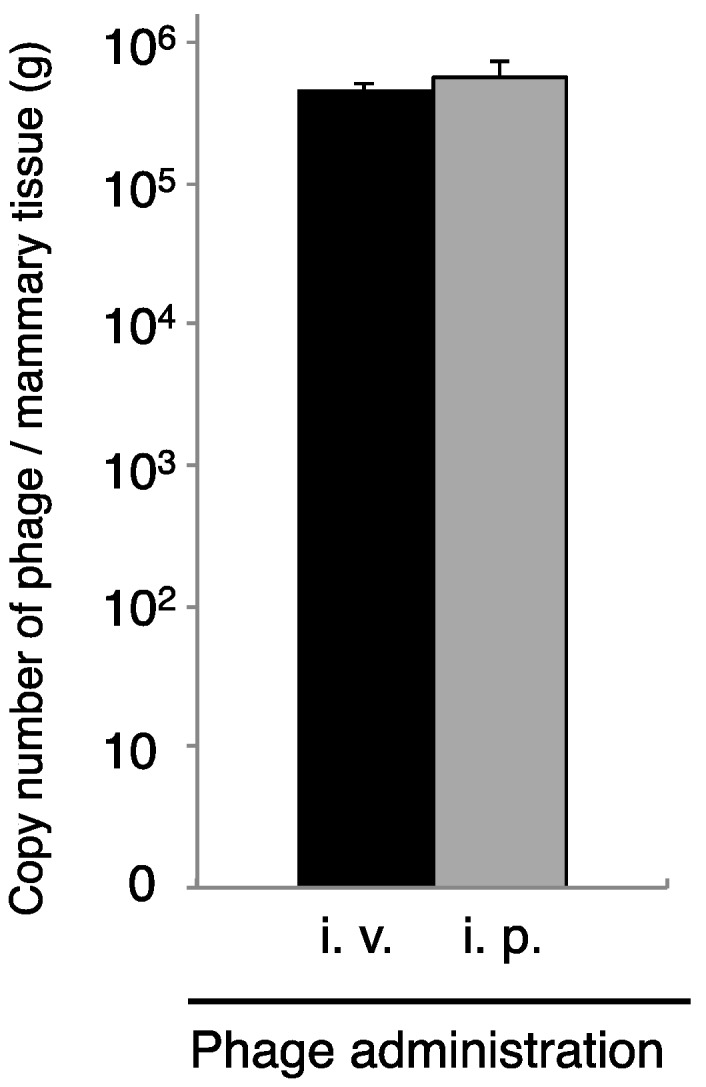
Transition of phage to the mammary gland by intravenous or intraperitoneal ΦSA012 administration. Mammary glands were separated at 30 min after phage administration (4 × 10^7^ PFU) through the intravenous (i.v., *n* = 13) or intraperitoneal (i.p., *n* = 14) route. Total DNA containing phage, host cells was purified from the mammary glands, and then phage copy numbers were determined by real-time PCR, using phage-specific primers. Bars indicate standard errors of the means. There was no statistically significant difference.

**Figure 4:**
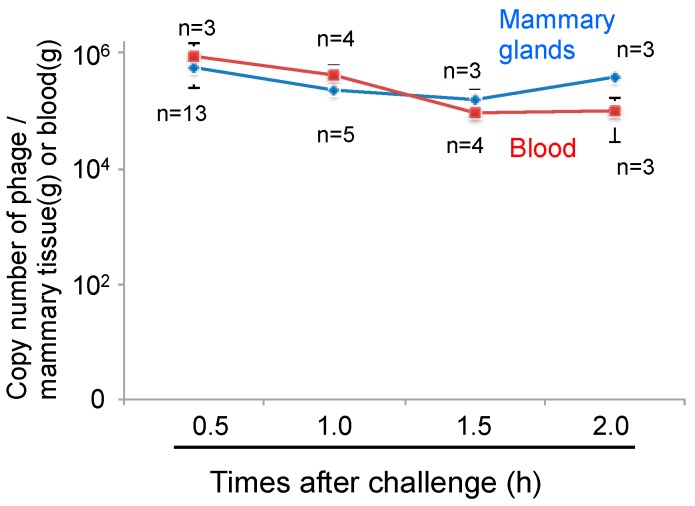
Kinetic analysis of phage distribution into the mammary glands through intraperitoneal administration. Mammary glands and blood were separated at 0.5 to 2.0 h after phage administration only (4 × 10^7^ PFU) through intraperitoneal route. Total DNA containing phage and host cells-derived DNA was purified from the mammary glands and blood and then phage copy numbers were determined by real-time PCR using phage-specific primers. Bars indicate standard errors of the means.

Phages cannot move by chemotaxis to infect bacteria in remote areas. Thus, diffusion of the phage into the area of infection is necessary for phage therapy. We next examined phage administration through the blood for phage delivery in the mammary gland. A high concentration of phage genomic DNA was detected 30 min after phage administration by both the intravenous and intraperitoneal routes (Figure 3). There was a no difference regarding phage transition to the mammary glands between intravenous and intraperitoneal phage administration (Figure 3). Next, kinetic analysis of phage ΦSA012 distribution in the blood and mammary glands through intraperitoneal administration was examined (Figure 4). Phage concentrations in both the blood and mammary glands decreased rapidly until 1.5 h after administration. At 2 h after phage administration, phage concentrations in the mammary gland increased. However, by 4 h, phage could not be detected in either the blood or mammary glands (data not shown). 

### 3.4. Therapeutic Efficacy of ΦSA012 Administered Intraperitoneally or Intravenously

**Figure 5:**
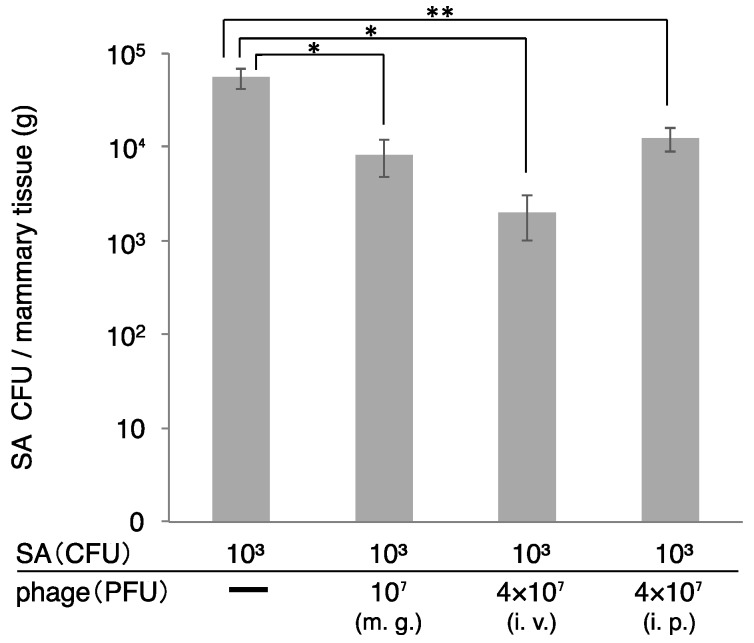
Therapeutic efficacy of ΦSA012 administered intraperitoneally or intravenously in a mouse model of *S. aureus* mastitis. SA003 (10^3^ CFU) was injected into the mammary gland and ΦSA012 was subsequently administrated to the mammary gland (10^7^ PFU), intraperitoneal (4 × 10^7^ PFU) or intravenous route (4 × 10^7^ PFU). At 2 days after administration of SA003 and ΦSA012, CFUs in the mammary gland were examined. m.g., mammary gland injection of ΦSA012; i.v., intravenous injection of ΦSA012; i.p., intraperitoneal injection of ΦSA012. All of the groups are *n* = 3. Bars indicate standard errors of the means. Statistically significant differences between control values and phage treatment values are denoted by asterisks (*, *p* < 0.01; **, *p* < 0.05 [determined using Tukey’s multiple-comparison test]).

*S. aureus* counts in the mammary gland were determined two days after administration of *S. aureus* and ΦSA012 (Figure 5). In this experiment, we changed the dose of host bacteria, because high dose of host bacteria produce high inflammations and tissue disruptions (Figure 1 and Figure 2). So, we changed the dose to the low dose of host bacteria (10^3^ CFU). *S. aureus* CFUs in the mammary gland decreased following phage administration by all the routes (mammary gland, intraperitoneal, and intravenous). Especially, *S. aureus* CFUs dramatically decreased in the group that received intraperitoneal and intravenous administration of ΦSA012. 

## 4. Discussion

The emergence of multidrug-resistant bacteria, such as MRSA, is a major concern for livestock and public health. *S. aureus* is also one of the most frequent causative agents of subclinical and clinical bovine mastitis, resulting in the high use of antibiotics [4]. Thus, there has been renewed interest in the use of phages as antimicrobial agents for infectious disease that is caused by drug-resistant bacteria. We previously reported the isolation of two novel *S. aureus* phages, designated ΦSA012 and ΦSA039, which were found to have a lytic effect on a broad range of *S. aureus* isolates obtained from mastitic cows [24]. In the present study, we initially examined the host range of ΦSA012 and ΦSA039 against *S. aureus* strains isolated from bovine mastitis. We then showed potential administration routes for effective phage therapy in a mouse mastitis model. 

ΦSA012 and ΦSA039 were found to have a lytic effect with broad range against 93 (40 genotypes) *S. aureus* strains isolated from milk of mastitic cows. The infection process of phages can be divided into the following steps: phage adsorption to the host, DNA injection into the host cell, DNA replication, assembly of phage particles, and lysis of the host cell. Adsorption of a phage to the host cell occurs by the interaction between a phage receptor on the bacterial surface and receptor-binding proteins (RBPs) in the tips of the tail fibers or tail spikes. This process is extremely specific; thus, RBPs determine the target bacteria for phage infection [32]. In the report, two proteins of ΦSA012, ORF103, and ORF105, were essential for phage binding to host cells. ORF103, which is a tail fiber component localized at the bottom of the baseplate, was shown to bind to α-*N*-acetylglucosamine (α-GlcNAc) in wall teichoic acids (WTAs) [32]. ORF105 was also shown to be an RBP that binds to WTAs. These findings suggest that most *S. aureus* strains analyzed in this study that cause mastitis may have α-GlcNAc on WTAs, enabling them to be targeted by ΦSA012 and ΦSA039.

Some groups have investigated phage therapy for bovine mastitis using mouse models [33,34] and bovine infection models [22,23]. Our mouse mastitis model demonstrated that ΦSA012 could reduce proliferation of *S. aureus* and inflammation in the mammary gland without causing any inflammation by phage administration only (Figure 1 and Figure 2). However, in these experiments, we did not determine phage counts from the tissue. Therefore, further examination regarding the effect of phage in the mastitis model is necessary. It was reported that the cure rate was 16.7% (3 of 18 quarters) in the phage-treated group of lactating Holstein cows, while none of the 20 saline-treated quarters were cured [22] Therefore, phage administration requires further optimization before use. 

One of the main challenges of phage therapy is phage delivery to areas of inflammation, because phages cannot move by chemotaxis to infect bacteria in remote areas and the udder is a large, complex structure. It was reported that the cure rate of bovine mastitis was 3 of 18 udder quarters (16.7%) in the phage-treated group [22]. Generally, mastitis caused by *S. aureus* is difficult to cure by antibiotic treatment [4]. In the present study, we examined phage delivery through the blood to inflamed areas in mammary glands. It is known that phage injected into the body simply spreads throughout the whole body and proliferates in the presence of host cells [15]. In the case of no host cells, phage may be quickly eliminated by macrophages. In the present study, phage administered without bacteria through the intravenous or intraperitoneal route could be detected in the mammary glands (approximately 1% of injected phage), and was promptly eliminated in the blood and mammary glands within 4 h after phage injection (Figure 4). Interestingly, phage copy numbers increased in the mammary gland at 2 h after phage injection, which may indicate the distribution and reallocation of phage from the blood to the tissue. At 1.5 h after phage administration, phage concentrations were inverted. We think that the spike of phage number in mammary gland is caused by reallocation from blood. Rapid elimination of phages in the absence of host cells has been also observed [15], suggesting that phage is removed by immune elimination. Therefore, one of the advantages of phage therapy may be the easy and quick removal of residual phage. Further studies are needed to understand the mechanisms of distribution and elimination of phage from the body.

Generally, mastitis therapy involves antibiotic administration through the teat. However, bacteria can hide in the udder, a large and complex structure, to escape from antimicrobial drugs. Moreover, it is challenging to deliver antibiotics to all areas of the udder, making mastitis treatment difficult. It has also been reported that milk components inhibit phage lytic activity [18]. Therefore, to effectively use phages for mastitis therapy, investigation of phage administration methods is necessary for on-site dairy production medicine. In the mouse mastitis model, phage administered through the intraperitoneal or intravenous route effectively decreased *S. aureus* counts in the mammary gland. These data demonstrated that phage could diffuse in the mammary gland through the blood and effectively kill bacteria. Phage administration through the blood is thought to be effective for tissue diffusion, but may result in immune elimination by the reticuloendothelial system. This is the most serious obstacle in the repetitive administration of phage through blood circulation. Merril et al. reported the development of phage mutants with the ability to avoid the reticuloendothelial system by using a serial passage technique in mouse, and these phage mutants could remain in the circulatory system for a longer period of time [16]. Although there are several issues to solve before application of phage therapy for bovine mastitis, phages are promising antibacterial agents for dairy production medicine. In the present paper, we demonstrated the potential of clinical use of phage in the mastitis mouse model, but Mouse and bovine are totally different. Therefore, we will need the experiments using bovine in the future.

## 5. Conclusions

We investigated the effect of phage therapy in a mouse mastitis model caused by *S. aureus* infection. *S. aureus* phages ΦSA012 and ΦSA039 showed broad host range against various *S. aureus* genotypes isolated from bovine mastitis. Using a mouse mastitis model, ΦSA012 reduced proliferation of *S. aureus* and accordingly inflammation in the mammary gland was relieved. Furthermore, intravenous or intraperitoneal phage administration reduced proliferation of *S. aureus* in the mammary glands. These results suggest that broad host range phages ΦSA012 is potential antibacterial agents for dairy production medicine.

## Figures and Tables

**Table 1:**
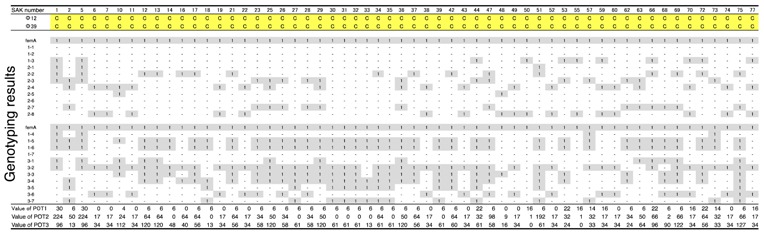
Host ranges of *S. aureus* phages, ΦSA012 and ΦSA039, against *S. aureus* isolated from mastitic cows in Kushiro.

Clear plaques (C, yellow boxes) indicate combinations resulting in the highest lysis activity in the spot test. 1 (gray boxes) indicates amplification with primers specific for *S. aureus* genome regions using the CicaGeneus Staph POT multiplex PCR kit. A difference in the value of POT indicates individual genotypes.

**Table 2:**
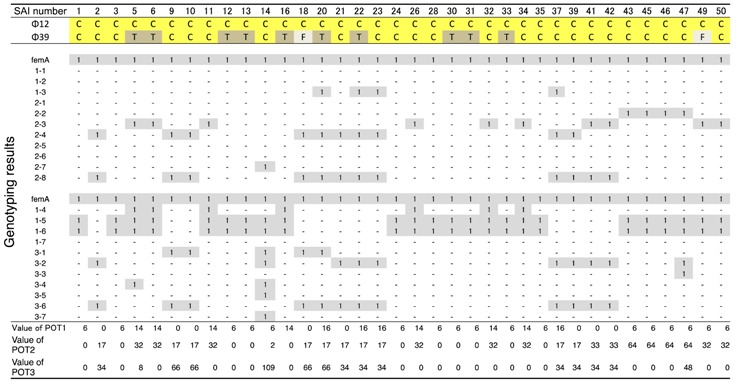
Host ranges of *S. aureus* phages, ΦSA012 and ΦSA039, against *S. aureus* isolated from mastitic cows in Ishikari.

Clear plaques (C, yellow boxes) indicate combinations resulting in the highest lysis activity, followed by turbid plaques (T, dark gray boxes); and, faint plaques (F, light gray bxes) in the spot test; 1 (gray boxes) indicates amplification with primers specific for *S. aureus* genome regions using the CicaGeneus Staph POT multiplex PCR kit. A difference in the value of POT indicates individual genotypes.

**Table 3:**
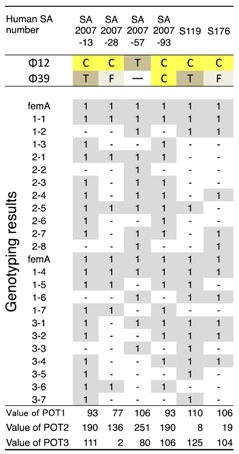
Host ranges of *S. aureus* phages, ΦSA012 and ΦSA039, against *S. aureus* methicillin-resistant *S. aureus* (MRSA) strains.

Clear plaques (C, yellow boxes) indicate combinations resulting in the highest lysis activity, followed by turbid plaques (T, dark gray boxes); and, faint plaques (F, light gray bxes) and no plaques (- in white box) in the spot test; 1 (gray boxes) indicates amplification with primers specific for *S. aureus* genome regions using the CicaGeneus Staph POT multiplex PCR kit. A difference in the value of POT indicates individual genotypes. MRSA strains are previously reported [8,31].

## Supplementary Materials

The following are available online at www.mdpi.com/2079-7737/7/1/8/s1. Table S1: Genotype and antimicrobial resistance types of MRSA strains.
