# Supplementary material for: Bacteriophage ΦSA012 Has a Broad Host Range against Staphylococcus aureus and Effective Lytic Capacity in a Mouse Mastitis Model
Source: Biology (Basel). 2018 Jan 9;7(1):8. doi: 10.3390/biology7010008 (PMC5872034; doi:10.3390/biology7010008)
Supplement: Supplementary file 1 [file biology-07-00008-s001.pdf]

biology

MDPI

## Supplementary Materials

**Table S1.** Genotype and antimicrobial resistance types of MRSA strains.

| No. | SCC*mec* | MLST | *spa* type | Antimicrobaial resistance |
|---|---|---|---|---|
| MRSA 2007-13 | II | NT | t002 | MPIPC, GM, KM, EM, LCM, OTC, ERFX |
| MRSA 2007-28 | II | NT | t1265 | MPIPC, KM, EM, LCM |
| MRSA 2007-57 | IV | NT | t008 | MPIPC, GM, KM, EM, LCM |
| MRSA 2007-93 | II | NT | t062 | MPIPC, KM, EM, LCM, OTC, CP, ERFX |
| MRSA VC39 Vet-1 | IV | ST30 | t021 | MPIPC, SM, KM, GM, EM |
| MRSA VC50 Vet-1 | IV | ST380 | t1852 | MPIPC, KM, GM, EM, CPFX |

NT indicates not tested. MPIPC; oxacillin, GM; gentamicin, KM; kanamycin, EM; erythromycin, LCM; lincomycin, OTC; oxytetracyclin, ERFX; enrofloxacin, CP; chloramphenicol SM; streptomycin, CPFX; ciprofloxacin
